# Spotlight environmental sustainability: a strategic priority for NICE

**DOI:** 10.1093/pubmed/fdac077

**Published:** 2022-08-18

**Authors:** Manuj Sharma, Sarah Walpole, Koonal Shah

**Affiliations:** Public Health Registrar, Health and Social Care, NICE; National Medical Director’s Clinical Fellow, Health and Social Care, NICE; Infectious Diseases and General Medicine SpR, North East of England; Associate Clinical Lecturer, Newcastle University; Associate Director, Science Policy and Research Programme, NICE

**Keywords:** environmental, health policy, health technology appraisal, sustainability, sustainable healthcare

## Abstract

This article provides the context for the ambition outlined in the the National Institute for Health and Care Excellence (NICE) 2021–2026 strategy to ‘lead globally on the potential to include environmental impact data in its guidance to reduce the carbon footprint of health and care’. Anthropogenic environmental changes pose a catastrophic risk to human health, with potential to widen national and global health inequalities. Recognising the fact that NICE guidance influences the way health and care is delivered and its consequent environmental impact, NICE has included environmental sustainability among its strategic priorities. This article outlines the work underway to meet this sustainability agenda at NICE.

## What’s new?

In recent years, the National Institute for Health and Care Excellence (NICE) has continued to expand its engagement with environmental sustainability and work to promote environmental sustainability in the health system.

NICE is identifying and supporting implementation of measures to assess and reduce the environmental impacts of NICE guidance and advice. A number of NICE products reference information on the environmental impact of health interventions to support more sustainable healthcare, including a medicine optimisation sustainability report, a patient decision aid for asthma inhalers and a medical technologies guideline.

Upcoming work will include formalising how environmental sustainability considerations are integrated into health technology evaluation and other NICE processes, developing the inhaler decision aid to enhance its accessibility and usefulness and running a programme of deliberative public engagement on sustainability.

NICE is also working to reduce its own organisational footprint. In 2022, NICE has been working to meet and surpass the ‘Greening Government Commitments’.

## Introduction

Anthropogenic environmental changes, including air and water pollution, climate change and biodiversity loss, pose potentially catastrophic risks to human health.^1^ Increased flooding, storms, heat stress, food and water insecurity, spread of disease vectors and worsening mental ill health are some of the direct and indirect consequences of these change.^2^ Those who are more socioeconomically deprived, have pre-existing health conditions or are older are more vulnerable to these effects, meaning climate change may widen existing health inequalities nationally and globally.^3^

The scale of the existential threat of climate change led the Intergovernmental Panel on Climate Change in 2018 to recommend that greenhouse gas emissions (GHGs) must reach net zero by 2050 to prevent a catastrophic temperature rise of 1.5°C.^4^ Understanding that health and care systems contribute 4–5% of these global GHGs,^5^ England's National Health Service (NHS) committed to achieving net zero by 2040, positioning itself as a vanguard of sustainability and an advocate that planetary and human health are inseparable.^1^ In January 2020, an ambitious sustainability programme was launched across the NHS under the banner of ‘Greener NHS’, a new organisation replacing the NHS Sustainable Development Unit.

Recognising that NICE guidance affects how the NHS operates and consequently its environmental impact, NICE laid out ambitions to support environmental sustainability in the NICE strategy 2021–2026.^6^^,^^7^

## NICE strategy 2021–2026 and sustainability

In the 2021–2026 NICE strategy, NICE outlines its ambition to ‘lead globally on the potential to include environmental impact data in its guidance to reduce the carbon footprint of health and care’.^7^ Recognising the public and political focus on environmental sustainability, and the need for collaboration across health system actors to achieve the NHS’s net zero ambition, NICE continues to strategize how it can best support the wider system on this agenda. This is expected to involve developing methods and processes to assess and reduce the environmental impacts (including GHGs, air pollution, water pollution and waste production) of implementing NICE recommendations.^6^ This requires consideration of how NICE will access, analyse and respond to data on environmental impacts alongside its core role of assessing data on health outcomes and economic impacts.

## Recent strategic and operational work at NICE on promoting environmental sustainability

Since this recent strategic commitment to support a sustainable health system, NICE has been working to identify and implement actions that it can take to support environmental sustainability across the health and care system. This requires collaboration to ensure coherence across different areas of NICE’s work, including overseeing methodological development work, and to share progress on NICE’s work to support sustainability.

NICE has previously demonstrated how implementation of its medicines optimisation guidelines on the safe and effective use of medicines in health and social care settings can also produce benefits from an environmental point of view.^8^ An embedded supportive environmental impact calculator within the report outlines how optimising medication usage leading to avoidance of hospital admissions could prevent over 110 000 tonnes of GHGs, save 179 million m^3^ of fresh water and prevent over 13 000 tonnes of waste generation. This is in addition to benefits associated with reducing an estimated £250–300 million of medicine that is wasted and goes unused each year.^8^

The responsibility to raise wider system awareness of environmental impacts of medication use extends to work NICE has been undertaking around use of inhalers for respiratory conditions. Inhalers are responsible for 3% of the NHS’s carbon footprint. A NICE patient decision aid included a question to enable patients to consider environmental impact when making a choice about which asthma inhaler they use.^9^

In guidance published in January 2022 (MTG65), ‘Sedaconda ACD-S for sedation with volatile anaesthetics in intensive care’, the medical technologies advisory committee considered environmental impact (based on data provided by the manufacturer), alongside clinical and cost effectiveness evidence. Sedaconda ACD-S is a system to capture and reduce the release of anaesthetic gases, which is important because volatile anaesthetic drugs are potent GHGs.^10^ As part of the committee assessment, evidence was considered indicating that Sedaconda-ACD-S could lead to lower consumption of other volatile sedatives and sedative agents and lower overall GHGs.^10^ A full evaluation of environmental impacts of Sedaconda-ACD-S was not completed however, and environmental impacts were not a driver of the committee’s decision to recommend the use of Sedaconda ACD-S. This example highlights an appetite to consider environmental sustainability and a need to develop standardised methods for environmental assessment and approaches to quality assure evidence on environmental sustainability.

In addition to supporting system sustainability across health and care, NICE is also working to reduce the environmental impact of its own activities given its reputational and leadership role globally. NICE adheres to the ‘Greening Government Commitments’ to:

- reduce carbon emissions
- minimise waste
- reduce water use
- make sustainable choices about procurement
- support biodiversity and nature recovery
- adapt to climate change and
- reduce the environmental impacts of digital working

NICE has an internal action-focused group engaging staff and working to reduce NICE’s environmental footprint in line with these commitments.

## Upcoming work: considering environmental sustainability in NICE’s processes and decisions

In line with its strategic commitment, NICE has commissioned an academic centre to explore possible approaches to incorporating environmental sustainability considerations in NICE’s methods and processes, including health technology evaluation. The academic centre has identified data sources and methods that can be used. This exploration has provided insights which may inform development of a framework for presenting and quantifying environmental impact information. Work is also underway to prepare for the implementation of such a framework, including deliberative public engagement to gain insights into public perceptions and preferences.

In response to the consultation at the scoping stage for the BTS/SIGN/NICE asthma guideline, which is due for publication in November 2022, stakeholders requested that NICE address environmental sustainability considerations. The importance of selecting the right inhaler device for each patient will be included in the guideline, and where devices are equally acceptable, environmental factors will be taken into account. The developers intend to recruit an expert on environmental issues related to respiratory care to support the committee.

During 2022, NICE is also undertaking focused projects to incorporate environmental considerations within specific activities. An important piece is a programme of deliberative public engagement, NICE Listens, which NICE will use to explore views on NICE’s role and priorities in the environmental sustainability space.

## Conclusion

In its 2021–2026 strategy, NICE has acknowledged the importance of assessing environmental impact in its guidance and the responsibility it has as a health leader to promote environmental sustainability. This article has highlighted how NICE is engaging with this agenda and contributing to the positive changes that are underway in the UK health system.

## Conflict of interest

S.W. is an unpaid Associate of the Centre for Sustainable Healthcare.

K.S. and S.W. are employed by the National Institute for Health and Care Excellence.

In his previous employments, K.S.' employers have received funding from a variety of sources, including pharmaceutical companies and the Association of the British Pharmaceutical Industry.

M.S. has nothing to declare.

## Notes

**Manuj Sharma**, Public Health Registrar

**Sarah Walpole**, Clinical Fellow

**Koonal Shah**, Associate Director
